# Forebrain Transcriptional Response to Transient Changes in Circulating Androgens in a Cichlid Fish

**DOI:** 10.1534/g3.119.400947

**Published:** 2020-04-10

**Authors:** Ana S. Félix, Sara D. Cardoso, António Roleira, Rui F. Oliveira

**Affiliations:** * ISPA - Instituto Universitário, 1149-041 Lisboa, Portugal,; ^†^ Instituto Gulbenkian de Ciência, 2780-156 Oeiras, Portugal, and; ^‡^ Champalimaud Research, 1400-038 Lisboa, Portugal

**Keywords:** Androgens, Challenge Hypothesis, Transcriptome, Sociogenomics, Brain

## Abstract

It has been hypothesized that androgens respond to the social interactions as a way to adjust the behavior of individuals to the challenges of the social environment in an adaptive manner. Therefore, it is expected that transient changes in circulating androgen levels within physiological scope should impact the state of the brain network that regulates social behavior, which should translate into adaptive behavioral changes. Here, we examined the effect that a transient peak in androgen circulating levels, which mimics socially driven changes in androgen levels, has on the forebrain state, which harbors most nuclei of the social decision-making network. For this purpose, we successfully induced transient changes in circulating androgen levels in an African cichlid fish (Mozambique tilapia, *Oreochromis mossambicus*) commonly used as a model in behavioral neuroendocrinology by injecting 11-ketotestosterone or testosterone, and compared the forebrain transcriptome of these individuals to control fish injected with vehicle. Forebrain samples were collected 30 min and 60 min after injection and analyzed using RNAseq. Our results showed that a transient peak in 11-ketotestosterone drives more accentuated changes in forebrain transcriptome than testosterone, and that transcriptomic impact was greater at the 30 min than at the 60 min post-androgen administration. Several genes involved in the regulation of translation, steroid metabolism, ion channel membrane receptors, and genes involved in epigenetic mechanisms were differentially expressed after 11-ketotestosterone or testosterone injection. In summary, this study identified specific candidate genes that may regulate socially driven changes in behavioral flexibility mediated by androgens.

Androgens are essential for reproduction. They influence morphology and physiology traits and have a pivotal role in the modulation of reproductive and also aggressive behaviors (Oliveira 2004). In turn, the social environment is known to modulate the circulating levels of androgens (Oliveira 2004, 2009). The Challenge Hypothesis has been proposed to explain androgen changes throughout the life history of an animal due to environmental (*e.g.*, photoperiod) and social cues (Wingfield *et al.* 1990; Goymann *et al.* 2007). This framework was initially suggested based on comparative data from bird species, but currently has been tested across all vertebrate taxa, including teleost fish (Oliveira 2004; Hirschenhauser and Oliveira 2006). According to this hypothesis, circulating androgens have their lowest levels in the non-breeding stage, while in the breeding season quite dynamic patterns are found. Herein, androgens vary between a breeding baseline (triggered for instance by day length) and a physiological maximum elicited by sexual interactions or aggressive confronts with conspecifics (Goymann 2009). So, the social modulation of androgens could be a proficient way of increasing androgens only when necessary, preventing extended high levels (and potentially harmful) in circulation. Indeed, despite the inherent benefits of androgens on the fitness of an animal (*e.g.*, spermatogenesis, secondary sex characteristics, mating success), elevated levels of androgens have relevant drawbacks. They interfere with paternal care and pair bonding, are energy-consuming and have been associated to immunosuppression and oncogenic effects (Wingfield *et al.* 2001; Oliveira 2004).

At a functional level, these socially driven changes in circulating steroid levels have been recognized to influence subsequent behaviors (Oliveira 2009). For instance, after a fight winner and loser effects (*i.e.*, animals which experience victory have a higher probability of winning subsequent matches and defeated animals are more likely to lose subsequent fights, respectively) have been described in many species, including teleost fish (Hsu *et al.* 2006). Interestingly, the winner effect seems to be mediated by androgens. In studies using the California mice (*Peromyscus californicus*) males that experience prior winnings have a transient increase of androgen levels (Oyegbile and Marler 2005) and are more aggressive in next fights (Trainor and Marler 2001). Furthermore, injecting androgens in castrated California mice males after winning a fight induces an increase in aggression in subsequent agonistic encounters in opposition to vehicle-injected males (Trainor *et al.* 2004). Moreover, in an African cichlid fish (Mozambique tilapia, *Orechromis mossambicus*) the administration of androgen antagonists blocks the winner effect (Oliveira *et al.* 2009). Altogether, these results demonstrate that androgens have a role in the integration of past social encounters, regulating aggression in future interactions (Wingfield 2005). Actually, it is the integration of information related to the social environment with internal features, such as previous social experiment and organism condition, which allows individuals to respond adaptively to changing social environments (Taborsky and Oliveira 2012; Oliveira and Oliveira 2014). Hormones, such as androgens, seem to be major players in this process acting as neuromodulators of neural circuits underlying social behavior (Oliveira 2009).

There is a growing body of literature that has identified a set of brain nuclei (aka Social Decision Making Network, SDMN), that together control social behavior (Goodson 2005; O’Connell and Hofmann 2011). The SDMN is constituted by interconnected core nodes whose concerted activity patterns correlate with the expression of distinct social behaviors, such as aggressive, mating or parental behaviors (*e.g.*, Newman 1999; O’Connell *et al.* 2012; Maruska *et al.* 2013). These brain nodes are mainly located in the forebrain and express sex-steroid and neuropeptide receptors, allowing the neuromodulation of the network by these hormones, including androgens (Goodson 2005; O’Connell and Hofmann 2011; Oliveira 2012). Moreover, the SDMN seems to be evolutionarily conserved across vertebrates (O’Connell and Hofmann 2012), and it has been extensively studied in non-mammalian species such as birds, reptiles and also teleost fish (*e.g.*, Teles *et al.* 2015; Roleira *et al.* 2017; Kabelik *et al.* 2018; Eswine *et al.* 2019). Thus, androgen neuromodulation of the neurogenomic sate of the SDMN is a candidate mechanism by which socially-driven transient changes in circulating androgens influence experience-driven behavioral flexibility. According to this hypothesis, changes in gene expression patterns of the SDMN should result in contrasting brain transcriptomes that would translate into different behavioral patterns (Cardoso *et al.* 2015), which highlights the relevance of transcriptomic studies in disclosing rapid shifts in the state of the neural network.

The aim of this study is to investigate the effect of a physiological and transient increase of androgens, that mimic the changes in androgen levels driven by social interactions, in the forebrain transcriptome. For this purpose, we characterized brain gene expression temporal patterns after pharmacologically manipulating animals’ hormonal states. We used Mozambique tilapia, an African cichlid fish with a lek-mating system (Fryer and Iles 1972). In this species, males exhibit two contrasting phenotypes. Dominants are usually larger, dark colored and establish territories to which they attract females and mate; while subordinates are faded color similarly to females and are not able to hold territories (Oliveira and Almada 1998). In the Mozambique tilapia, androgens influence social behavior and also respond to the social environment (Oliveira 2009). In this study, we injected dominant male fish either with 11-ketotestosterone (KT) or testosterone (T) and compared with a group injected with vehicle solution. We focused on the forebrain because the expression of androgen receptors in the forebrain of teleost fish is broad (*e.g.*, *P. notatus*, Forlano *et al.* 2010; *Carassius auratus*, Gelinas and Callard 1997; *A. burtoni*, Harbott *et al.* 2007; Munchrath and Hofmann 2010). We collected samples at different sampling times (30 min and 60 min) after treatment injection to detect transient changes that occur in the gene expression patterns by using the RNAseq technique. The importance of characterizing the temporal dynamics of brain activity in behavioral genomics has been highlighted by some authors (Rittschof and Hughes 2018; Renn and Aubin-Horth 2019). Our hypothesis is that the genomic response would be sustained since it is known that a cascade of events occurs in response to relevant stimuli, beginning with the activation of pre-existing proteins (*e.g.*, phosphorylation of cAMP response element-binding protein) that elicit the rapid expression of immediate early genes which in turn act as transcription regulators of genes later expressed (Clayton 2000, 2013; Cardoso *et al.* 2015). As a consequence, we expected the existence of multiple waves of gene expression that would be extended from minutes to hours and wanted to determine if there is a unitary genomic response or either different sets of genes are expressed at distinct time stages.

## Materials and Methods

### Animals and housing

*O. mossambicus* adult males from a stock held at ISPA were used in this experiment. Fish were maintained in glass tanks (120 × 40 × 50 cm, 240 l) with a fine gravel substrate. Tanks were supplied with a double filtering system (gravel and external biofilter) and constant aeration. Water quality was analyzed twice per month for nitrites (0.2–0.5 ppm), ammonia (<0.5 ppm, Pallintest kit) and pH (6.0 - 6.2). Fish were kept at a temperature of 26 ± 2°, a 12L:12D photoperiod, and fed with commercial cichlid floating sticks. Ninety-nine focal dominant males (weight: mean body mass ± SEM: 44.64 g ± 1.00 g; mean standard length ± SEM: 11.23 cm ± 0.12 cm; age: 2.5 - 3 years old) were used in this study. There was no difference in body size or weight between treatments (see below; t(18) = 1.767, *P* = 0.094).

Males’ social status was monitored several times per week and territorial males were identified based on nuptial black coloration and exhibition of reproductive behavior, including territory defense and digging of a spawning pit in the substrate, for at least 1 week (Oliveira and Almada 1996).

### Experimental setup

Subjects were lightly anesthetized (MS-222, Pharmaq; 300 ppm) to be weighted and measured and then individually housed in experimental tanks. Each experimental tank (50 × 25 × 30 cm, 40 L) had opaque lateral walls to prevent male’s visual contact with adjacent tanks. After 1 week of isolation, focal males were arbitrarily assigned to one of the following treatments: intra-peritoneal (i.p.) injection with (1) 11-ketotestosterone (KT-treated group); (2) testosterone (T-treated group); or (3) vehicle (V-treated group). Focal males were injected, returned to experimental tanks and sampled 15, 30 or 60 min after injection to collect blood (sample size of 8-12 per group) and/or brain. A control group, similarly isolated for one week but not injected, was sampled for blood to measure baseline androgen levels. To reduce hormonal fluctuations associated with natural circadian rhythm, the experiment was conducted in the morning.

11-ketotestosterone dose (Steraloids, 0.02 μg/g BW) was selected based on a pilot experiment where three different doses were tested in castrated male fish. We selected the dose that produced a significant physiological increase in circulating levels (Figure S1) similar to the one observed for this species in male-male interactions (Félix *et al*. 2020). Testosterone (Steraloids) concentration used was also 0.02 μg/g based on a previous study (Huggard *et al.* 1996: in this experiment, by using goldfish, *Carassius auratus*, several testosterone dosages, at physiological levels, were administered *in vivo* and resulted in the stimulation of gonadotropin subunit gene expression in the fish pituitary proving to be involved in steroidogenic regulation; 0.02 μg/g was the lowest concentration used). Stock hormones were dissolved in 100% ethanol to a concentration of 0.5 mg/ml and then diluted in saline solution (0.9% sodium chloride) until their final concentration. Vehicle solution consisted in 0.05% ethanol in saline solution.

### Blood sampling

Males were anesthetized (MS-222, Pharmaq; 450 ppm) and blood was collected from the caudal vein using heparinized 25-gauge needles. Blood sampling always took place within 4 min of the induction of anesthesia to prevent possible effects of handling stress on steroids levels (Foo and Lam 1993). Blood samples were centrifuged (10 min, 3000 *g*, 4°) and plasma was stored at – 20° until further processing.

### Hormone assays

11-ketotestosterone (KT) and testosterone (T) free steroids were extracted from plasma samples by adding diethyl-ether (Merck). Samples were then agitated for 20 min, centrifuged (5 min, 163 *g*, 4°) for phase separation and kept at -80° for 15 min to freeze the water phase and separate the ether fraction (containing the free steroid). This process was repeated twice to obtain higher extraction efficiency. Ether fraction was evaporated with a speedvac (Savant SC1101) and the dried organic phase was re-suspended in phosphate buffer. Steroid concentrations were measured by radioimmunoassay using a T antibody from Research Diagnostics Inc (#WLI-T3003, rabbit anti-testosterone). The antibody used for KT was kindly donated by D. E. Kime and the corresponding specificity table was published in Kime and Manning (1982). The reactive marker used for T was from Amersham Biosciences ([1,2,6,7-3H] Testosterone, #TRK402-250 µCi) while KT marker was produced in-house from marked cortisol (Kime and Manning 1982). Inter-assay variabilities were 5.3% for KT and 8.2% for T. Intra-assay variation coefficients were 2.4%, 2.1% and 7.6% for KT and 8.9%, 8.2% and 4.5% for T.

### Tissue processing and RNA extraction

We randomly selected 5 focal males for brain analysis from each one of the following experimental treatments: KT-group (2 sampling time points: 30 min and 60 min), T-group (2 sampling time points: 30 min and 60 min) and V-treated group (2 sampling time points: 30 min and 60 min). In total we killed 30 individuals, with an overdose of MS-222 (Pharmaq; 800 ppm). These sampling times take in consideration the time course of the socially driven androgen response in *O. mossambicus* which shows two peaks, an earlier one at 5-15 min and a late one at 60-90 min, and aim to assess the effects of the early androgen peak on brain state. Although no data are available for *O. mossambicus* on the time lag between the circulating and brain androgen peak in response to social interactions, it is known from other species that steroids in the brain peak 20-30 min later than in plasma (Droste *et al.* 2008; Remage-Healey *et al.* 2008). After sectioning of the spinal cord, forebrain area (olfactory bulbs, telencephalon and diencephalon) was dissected under a stereomicroscope (VWR SZB200) and collected in 500 µl of Qiazol lysis buffer (RNeasy Lipid Tissue Mini Kit, Qiagen). Samples were stored at -80° until RNA extraction. Total RNA was extracted using RNeasy Lipid Tissue Mini Kit (Qiagen) with some protocol adjustments. Briefly, samples were homogenized with a pellet pestle motor (Kontes) and added 100 µl of chloroform. Incubation times were increased in order to maximize RNA recovery and in the end samples were diluted in 50 µl of RNase-free water. DNase digestion was performed to guarantee samples free of DNA contamination. RNA quantity was assessed using a Nanodrop spectrophotometer (Thermo Scientific) and RNA integrity was confirmed using Bioanalyzer (Agilent). RNA was stored at -80° until processing.

### Library preparation, RNA sequencing and reference genome mapping

cDNA was generated with SmartSeq2 protocol (Picelli *et al.* 2014) and libraries were prepared with an optimized Nextera protocol (Baym *et al.* 2015).

RNA libraries of the 30 samples were pooled and sequencing was performed by the Centre for Genomic Regulation (CRG, Barcelona, Spain). cDNAs were amplified according to the Illumina RNA-Seq protocol and sequenced in three lanes using the Illumina HiSeq 2500 v4 system as paired-end 75-bp reads so that 200-300 million reads per lane (*i.e.*, 20-30 Mio reads/sample) could be achieved.

Quality of the data were checked with FASTQC software 0.11.7 (Andrews 2010). Cutadapt 1.18 (Martin 2011) was used to remove low quality reads (quality-cutoff set to 20) and adapter sequences keeping only paired end-reads having a minimum length of 30 bp. Clean reads were mapped onto the Nile tilapia, *Oreochromis niloticus*, reference genome (Oreochromis_niloticus.Orenil1.0.92) using Hisat2 2.1.0 (Kim *et al.* 2015). Quality control of alignments was ascertained with Qualimap 2.2.1 (Okonechnikov *et al.* 2016) and the table of counts was generated with FeatureCounts 1.6.1 (Liao *et al.* 2014). The RNAseq produced a total number of clean reads that ranged between 10.06 and 29.2 million reads. About 8.56 to 26.16 million reads were mapped onto the genome.

### Data analysis

#### Hormone analysis:

Normality of steroid data were tested by analyzing skewness and kurtosis values (Kline 1998) and running Shapiro-Wilk tests. Hormone variables were log-transformed to meet parametric assumptions. Outliers were identified using a generalized extreme studentized deviate procedure (*P* = 0.05, maximum number of outliers set to 20% of the sample size) and removed from data. Homoscedascity was confirmed with Levene’s test. Hormone levels (KT, T) were analyzed using planned comparisons to compare steroid levels between each time-point (15, 30 and 60 min) and the baseline (no-injection group) for each treatment (KT-, T- or V-treated groups). P-values were adjusted using the Benjamini and Hochberg (1995) procedure. Effect sizes were computed for planned comparisons (Cohen’s d). Statistical analysis was performed using R (R Core Team 2015) and STATISTICA v.10 (StatsoftInc).

#### Differential gene expression analysis:

Gene counts were imported to R, and edgeR 3.18.1 package was used for gene expression analysis (Robinson *et al.* 2010). We filtered genes with very low levels of expression levels (≤ 1 CPM) and retained genes expressed in at least 3 samples. An exploratory analysis was performed by Principal Component Analysis (PCA) to check relative similarities among replicates. One of the samples from the V-treated group was identified as an outlier and excluded from further analyses (Figure S2).

Differentially expressed (DE) genes were determined for each experimental group (KT- and T-treated groups) using the V-treated group as a reference. Counts were normalized using the TMM normalization method and the generalized linear model (GLM) likelihood ratio (LR) test for significance was implemented in edgeR (Robinson *et al.* 2010) for each time point separately (30 min and 60 min). P-values were adjusted for multiple testing using false discovery rates (FDR) with the Benjamini and Hochberg (1995) procedure, and genes with an FDR < 0.1 were considered as being differentially expressed. For visualization of the global expression patterns of DE genes among treatment groups, a hierarchical clustering analysis was performed for each time point. The reliability of the hierarchical cluster was assessed by 1,000 bootstrap resampling of the expression values using the R package pvclust 2.0 (Suzuki and Shimodaira 2006). Heatmaps were produced with the hclust function in R, adapted to produce a hierarchical clustering of Z-transformed expression values using Euclidean distance with complete linkage. A PCA was also conducted to cluster samples by groups using DE genes (Figures S3 and S4).

Tilapia gene annotation and gene ontology terms were obtained from the ENSEMBL BIOMART database. GO term enrichment for genes detected as differentially expressed were evaluated in GOstats v2.42.0 (Falcon and Gentleman 2007), using a ‘conditional’ hypergeometric test with a P-value < 0.05. This method accounts for the hierarchical relationships of GO terms, and hence, a formal correction for multiple testing cannot be applied due to the implicit dependence between neighboring GO terms, which do not comply with the independence of tests needed for correction of the p-values. The relative contribution of GO enrichment data in terms of GO classes they represent was visualized using the GO slim vocabulary and the web tool CateGOrizer (Zhi-Liang *et al.* 2008).

The R package GeneOverlap 1.22 (Shen and Sinai 2019) was used to assess the significance of the overlap between the DEG lists obtained for the KT-group and the T-group.

### Ethics statement

Experimental procedures used in this study were conducted in accordance with the institutional guidelines for the use of animals in experimentation and were approved both by the internal Ethics Committee of ISPA and by the National Veterinary Authority (Direção Geral de Alimentação e Veterinária, Portugal; permit number 0421/000/000/2013).

### Data availability

Raw sequencing data were deposited in BioProject portal at NCBI (BioProject ID PRJNA591471; http://www.ncbi.nlm.nih.gov/bioproject/591471). Table S1 includes each sample library information such as total number of reads and mapped reads. Table S2 contains gene counts generated with FeatureCounts. Full gene lists and DEG gene lists determined for the KT-treated group and using the V-treated group as a reference are available in Table S3 and Table S4, respectively for the 30 min and 60 min time points. Full gene lists and DEG gene lists determined for the T-treated group and using the V-treated group as a reference are available in Table S5 and Table S6, respectively for the 30 min and 60 min time points. Table S7 contains the list of DEG genes which overlap between the KT-group and T-group, for the 30 min time point. Table S8 contains the results for the GO term enrichment for genes detected as differentially expressed. Supplemental material available at figshare: https://doi.org/10.25387/g3.11987283.

## Results

### Hormone levels

**Figure 1:**
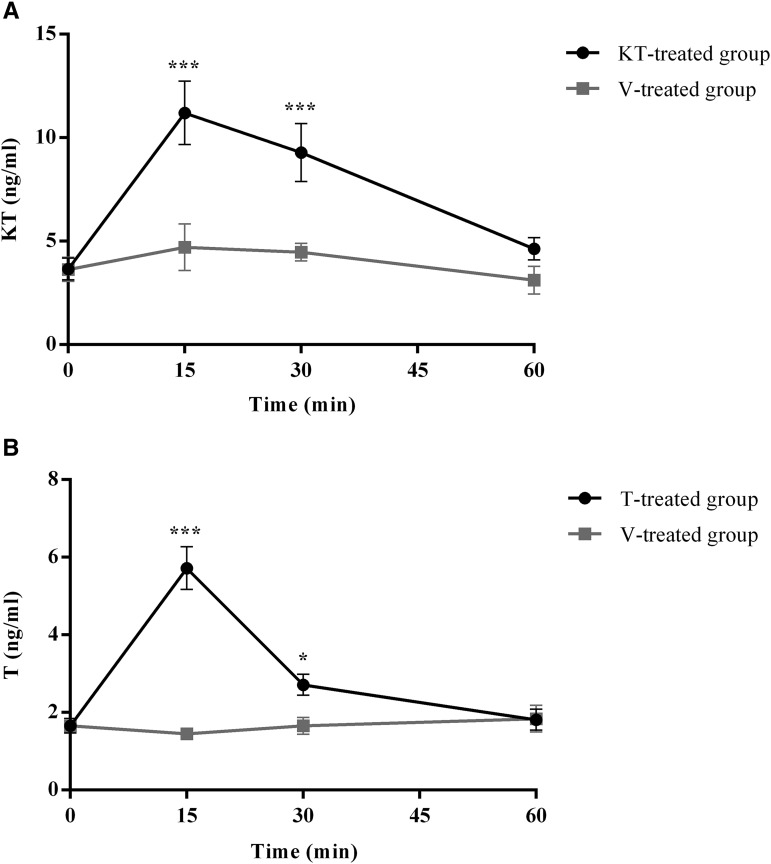
Temporal pattern of androgen circulating levels of fish injected with vehicle (V-treated group), 11-ketotestosterone (KT-treated group) or testosterone (T-treated group). Sample size for each point: 8-11 individuals. Values are mean ± standard error of the mean (SEM). A. 11-ketotestosterone (KT) levels of V and KT-treated groups; B. testosterone (T) levels of V and T-treated groups; * *P* < 0.05; *** *P* < 0.001.

The levels of KT and T changed significantly with time and treatment (Table 1). Androgen treated fish (either KT-treated or T-treated) had a significant increase above baseline of the injected androgen 15 min and 30 min but not 60 min after administration (Table 1, Figure 1). There were no differences in either KT or T in fish injected with vehicle (V-treated group) (Table 1, Figure 1).

**Table 1:** Effect of time and treatment (KT, T or vehicle) on circulating hormone levels. Planned comparisons and effect sizes between the baseline and the other time points for each treatment

Comparisons	Planned Comparisons			Vehicle group (V)		
	Androgen-treated group					
	t	p	d	t	p	d
***KT***						
0 min *vs.* 15 min	**−4.681**	**<0.0001**	**2.089**	−0.3361	0.7378	0.104
0 min *vs.* 30 min	**−3.722**	**0.001**	**1.857**	−1.362	0.355	0.514
0 min *vs.* 60 min	−1.126	0.396	0.578	0.7978	0.513	0.101
***T***						
0 min *vs.* 15 min	**−6.482**	**<0.0001**	**3.449**	0.6376	0.5258	0.416
0 min *vs.* 30 min	**−2.715**	**0.025**	**1.486**	0.2003	0.9461	0.339
0 min *vs.* 60 min	−1.400	0.332	0.212	0.0678	0.9461	0.200

11-ketotestosterone (KT); testosterone (T); t-test estimate; d: effect size estimate (Cohen’s d); p: p-value after multiple comparison adjustment; statistically significant values are in bold.

### Forebrain genomic response at 30 min after androgen administration

**Figure 2:**
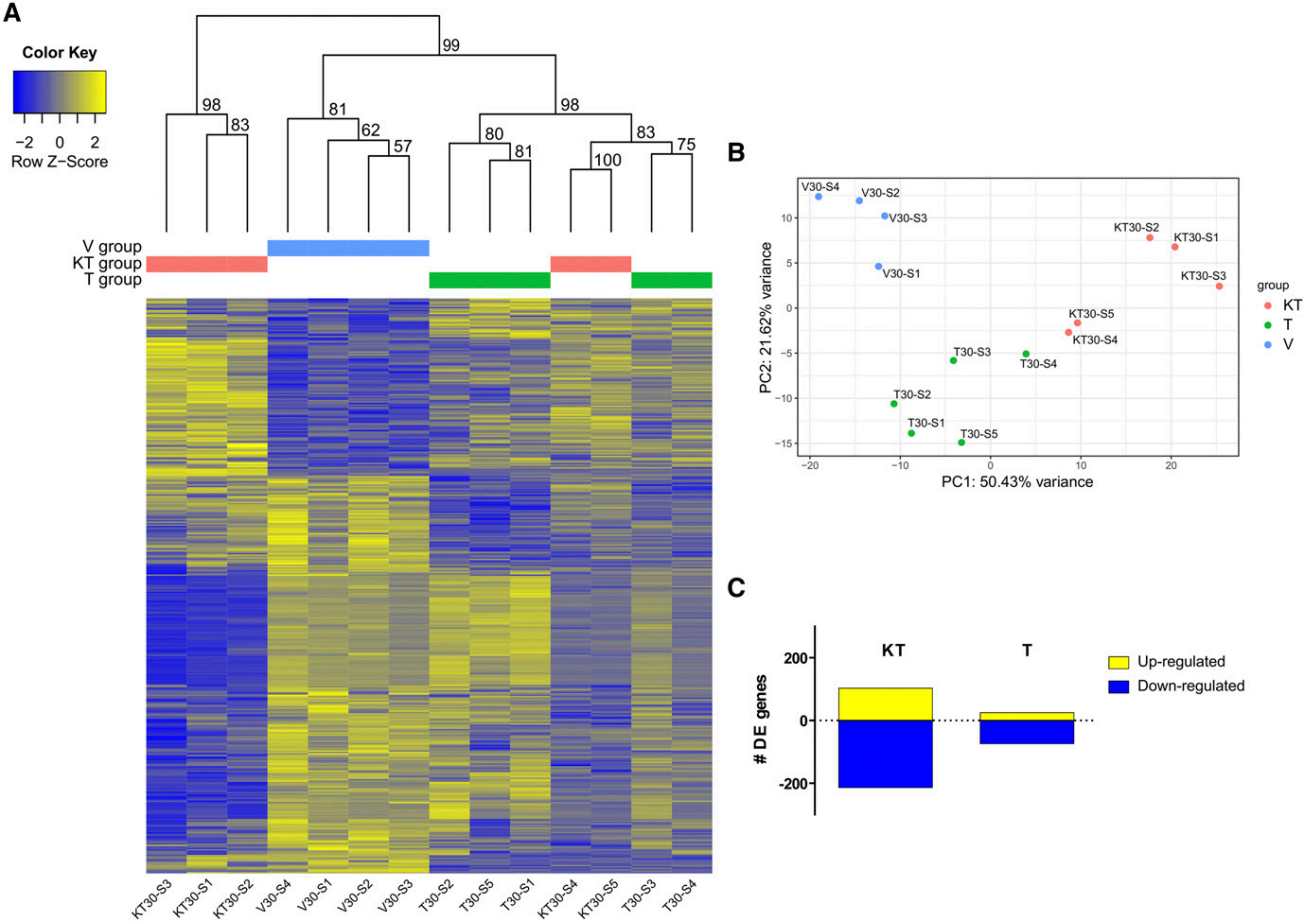
Differences in forebrain gene expression patterns of fish injected with vehicle (V-treated group), 11-ketotestosterone (KT-treated group) or testosterone (T-treated group) at 30 min post-injection: A. Heatmap of differentially expressed genes. Intensity of color indicates relative expression levels of each gene (rows) in each treatment (columns), with blue representing downregulated transcripts and yellow upregulated transcripts. For each cluster obtained with hierarchical clustering, unbiased p-values (value between 0 and 1 but here in %) can be seen above the heatmap. These values were calculated via multiscale bootstrap resampling, indicating how strong the cluster is supported by data. B. Principle Component Analysis (PCA) of DE genes of fish from the three treatment groups. C. Number of differentially expressed genes of fish injected with 11-ketotestosterone (KT-treated group) or testosterone (T-treated group) using a vehicle group (V-treated group) as a reference group.

A total of 319 differentially expressed (DE) genes was observed in the KT-treated group compared with the V-treated group, of which 104 were up-regulated and 215 down-regulated (Figure 2C, Table S3). In the T-treated group, 101 DE genes were found compared with the V-treated group, of which 26 were up-regulated and 75 down-regulated (Figure 2C, Table S5). Eighteen genes were DE both in the KT- and T-treated groups relative to the V-treated group (Table S7). The overlap between the two DE lists (KT-group *vs.* T-group) considering the whole set of genes in the genome was small but statistically significant (*P* < 0.001, OR = 19.2), even though the similarity between the two lists is very low (Jaccard similarity index = 0.0).

Hierarchical clustering shows that although all V-treated individuals clustered together according to their DE genes, KT-treated and T- treated individuals did not cluster according to their DE genes (Figure 2A). Principal component analysis showed that that the treatments tend to separate, with the first component explaining 59.4% of the variance and separating the 3 treatments (Figure 2B), whereas the second component describes 18.5% of the variance in DE genes and allows separation between the V-treated and the androgen treated groups.

**Figure 3:**
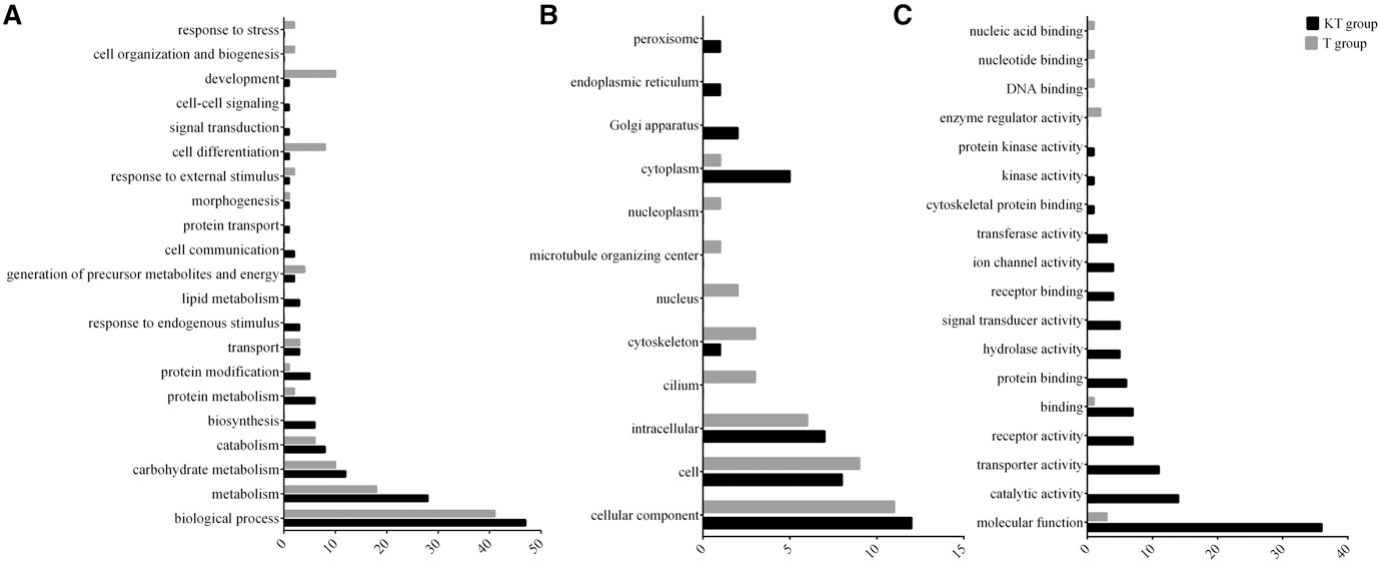
Differences between KT-treated and T-treated fish at 30 min post-injection in the representation of DE genes in the gene ontology (GO) classes for each ontology: A. Biological Process, B. Cellular Component and C. Molecular Function. Enriched GO terms were obtained for upregulated transcripts for each treatment group and mapped to a total of 127 GO slim ancestor terms with CateGOrizer.

**Figure 4:**
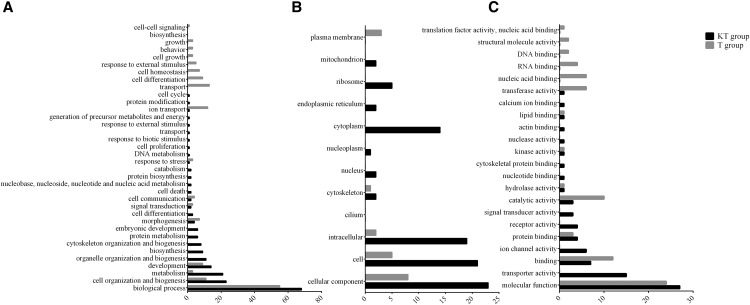
Differences between KT-treated and T-treated fish at 30 min post-injection in the representation of DE genes in the gene ontology (GO) classes for each ontology: A. Biological Process, B. Cellular Component and C. Molecular Function. Enriched GO terms were obtained for downregulated transcripts for each treatment group and mapped to a total of 127 GO slim ancestor terms with CateGOrizer.

The GO analysis (Table S8) found different biological processes, cellular components and molecular functions enriched by DE genes for KT- and T-treated groups. For up-regulated DE genes, KT-treated group had enrichment of processes related to metabolism, carbohydrate metabolism, cell and catalytic and transporter activity, while T-treated group had a predominant enrichment of processes related to metabolism, development and cell differentiation (Figure 3). The vast majority of down-regulated DE genes of the KT-treated group were associated to metabolism and cell organization, cell and intracellular and binding and catalytic activity, while for the T-treated group, these genes were associated to transport, ion transport, cell and transporter activity (Figure 4).

### Forebrain genomic response at 60 min after androgen administration

**Figure 5:**
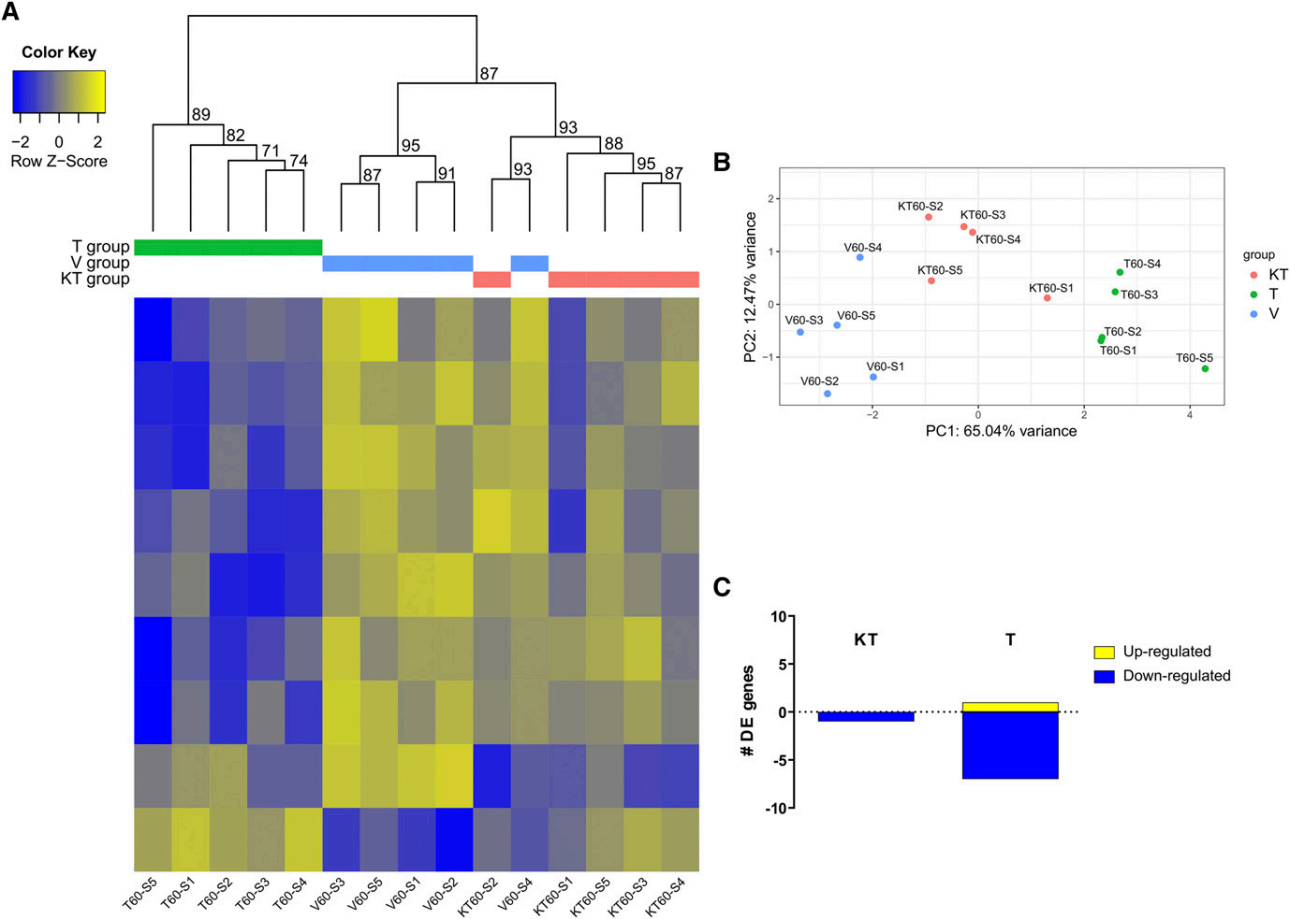
Differences in brain expression patterns of fish injected with vehicle (V-treated group), 11-ketotestosterone (KT-treated group) or testosterone (T-treated group); sampling time point of 60 min. A. Heatmap of differentially expressed genes. Intensity of color indicates relative expression levels of each gene (rows) in each treatment sample (columns), with blue representing downregulated transcripts and yellow upregulated transcripts. For each cluster obtained with hierarchical clustering, unbiased p-values (value between 0 and 1 but here in %) can be seen above the heatmap. These values were calculated via multiscale bootstrap resampling, indicating how strong the cluster is supported by data. B. Principle Component Analysis (PCA) of DE genes of fish from the three treatment groups. C. Number of differentially expressed genes of fish injected with 11-ketotestosterone (KT-treated group) or testosterone (T-treated group) using a vehicle group (V-treated group) as a reference group.

Only 1 differentially expressed (DE) gene was observed in the KT-treated group compared with the V-treated group, which was down-regulated (Table S4). In the T-treated group, 8 DE genes were found compared with the V-treated group, 1 up-regulated and 7 down-regulated (Table S6). The DE gene observed in the KT-treated group was not present in the list of DE genes obtained for the T-treated group. Hierarchical clustering shows that although all except two individuals clustered following their treatment according to their DE genes (Figure 5A). Principal component analysis shows that that the groups tend to separate, with the first component explaining 65.0% of the variance and separating all groups. The second component describes 12.5% of the variance of DE genes (Figure 5B).

**Figure 6:**
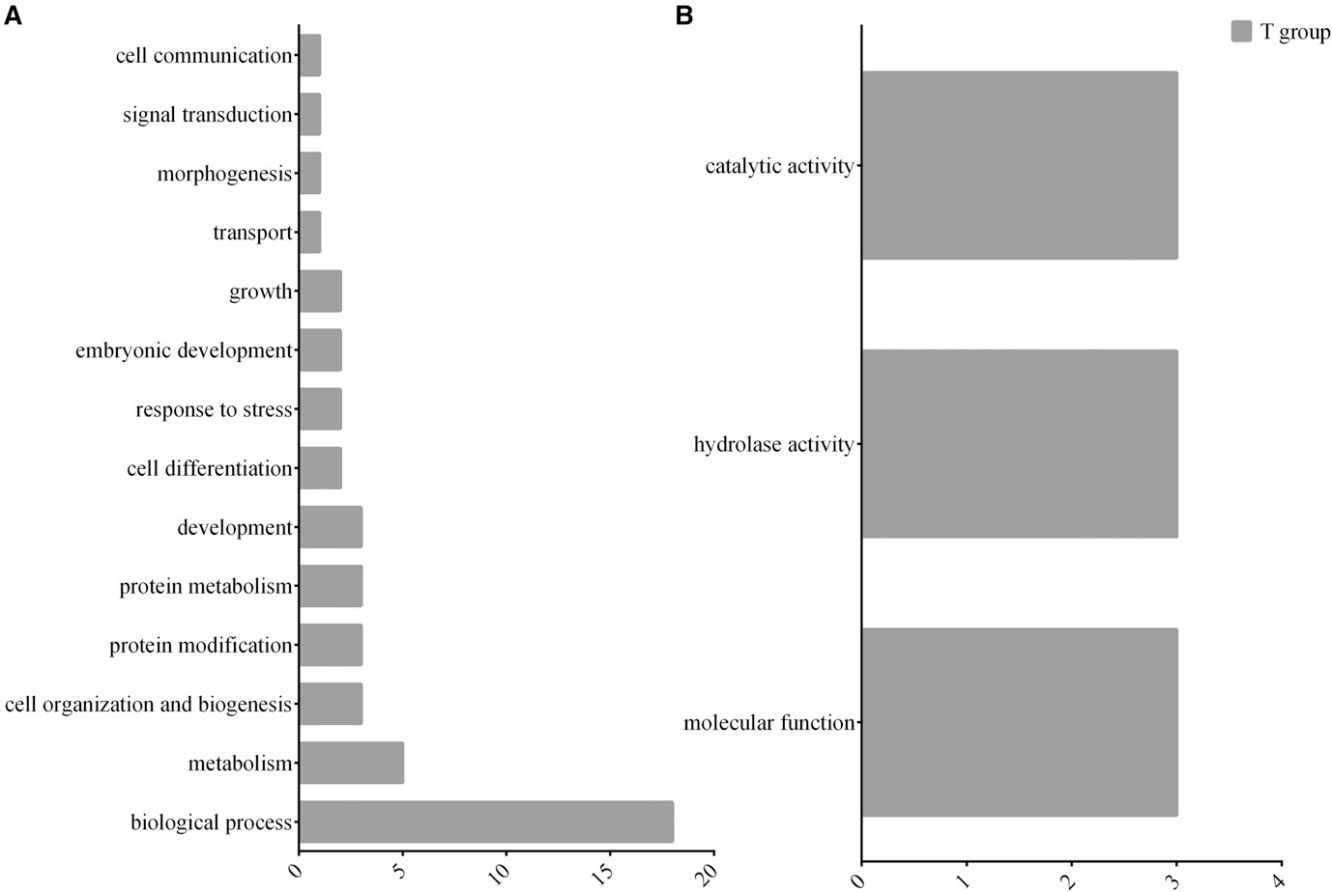
Representation of DE genes in the gene ontology (GO) classes for each ontology for T-treated fish at 60 min post-injection: A. Biological Process and B. Molecular Function. Enriched GO terms were obtained for downregulated transcripts and mapped to a total of 127 GO slim ancestor terms with CateGOrizer.

We did not perform the GO analysis for the KT-treated group since only 1 DE gene was observed in this group. For the same reason, the GO analysis for the T-treated group was performed only to the set of down-regulated genes (Table S8) and identified that the vast majority of down-regulated genes of the T-treated group were associated to metabolism and catalytic/hydrolase activity (Figure 6).

## Discussion

Our results show that a physiological and transient increase of circulating androgens, which mimics the transient androgen response to social interactions, induces significant changes in the pattern of forebrain gene expression in Mozambique tilapia territorial males. Individuals injected with KT experienced a transient increase of KT levels and had a higher number of genes differentially expressed relative to vehicle-treated fish, than individuals injected with T which also had a transient increase of T levels. Moreover, in both androgen treatments there were more genes differentially expressed in the forebrain 30 min after the injection than in 60 min after the injection. Together these results indicate that transient changes in circulating KT have a higher impact in changes in the forebrain transcriptome, which may underlie adaptive behavioral responses to social challenges.

A growing body of research has adopted genomic scale gene expression studies to unravel the brain mechanisms associated to social interactions [*e.g.*, mating behavior, (Lawniczak and Begun 2004); affiliative interactions: (Shpigler *et al.* 2019); agonistic interactions: (Oliveira *et al.* 2016); social eavesdropping: (Lopes *et al.* 2015); mate choice: (Cummings *et al.* 2008)]. Specifically the transcriptomic response to social challenges posed by brief territorial intrusions have been described in a comparative manner across taxa [*i.e.* in the house mouse (*Mus musculus*), the threespined stickleback, *Gasterosteus aculeatus*, and in the honey bee, *Apis mellifera*] and genes related to hormones are commonly affected (Rittschof *et al.* 2014). On the other hand, the effect of chronic exposure to androgens on the brain during development or in adulthood has been documented (Peterson *et al.* 2013; Ghahramani *et al.* 2014). However, to our knowledge, the specific effect of an acute and transient peak of androgens, like the one observed in response to social interactions, on the brain, has not been investigated.

In teleost fish, KT is considered the main circulating androgen since it has a higher impact than T on spermatogenesis, secondary sex characters and sexual behavior (reviewed by Borg 1994). In several teleost species, including the Mozambique tilapia, KT responds to social interactions, contrary to T (*e.g.*, Hirschenhauser *et al.*, 2004, Oliveira *et al.*, 1996). The present study confirms KT as more effective than T in producing significant changes in the brain transcriptome. Interestingly, in this study both androgens are shown to induce the differential expression of several (>100) genes in the brain of the Mozambique tilapia. However, different sets of genes are DE-expressed in KT and T treated fish.

For the KT-treated group (30 min sampling time point), several genes involved in the regulation of translation (*e.g.*, ribosomal proteins) or steroid metabolism (dehydrogenase/reductase, cholesterol 24-hydroxylase) were differentially expressed but many other genes were affected. For instance, kisspeptin-2, a gene known to regulate the hypothalamus-pituitary-gonads (HPG) axis, by modulating gonadotropin secretion (Nile tilapia, Park *et al.* 2016) and consequently androgen release, was down-regulated. Likewise, the estrogen receptor membrane was downregulated supporting evidence from previous observations (*e.g.*, androgen treatment of castrated rats reduces expression levels of estrogen receptor in the ventrolateral part of the ventromedial nucleus hypothalamus; Simerly and Young 1991), and confirming that, in the Mozambique tilapia, androgens also affect estrogen receptor expression and seem to modulate the hormonal responsiveness of estrogen receptor containing neurons. On the other hand, the immediate early gene *c-fos*, a gene known to orchestrate the transcriptomic responses to social challenges in both mice and zebrafish (Malki *et al.*, 2016) by acting as a transcription factor that regulates the MAPK signaling pathway involved in neurosecretion and structural plasticity (Clayton 2000), was up-regulated. Moreover, it is known that steroids can induce cell changes in a question of minutes or even seconds through nongenomic mechanisms, typically involving intracellular second messengers (mostly calcium changes) and signal transduction cascades (Michels & Hoppe 2008). For instance, studies have described the activation of membrane receptors, hormone-binding globulin receptors, protein kinases or the regulation of voltage- and ligand-gated ion channels and transporters within these mechanisms (reviewed in Michels and Hoppe 2008) that can also affect gene transcription (Foradori *et al.* 2008). Accordingly, in this study, we detected the up-regulation of ion channel membrane receptors (glycine receptors, *glra2*, *glrbb*; G-protein coupled receptors, *gprc5bb*; glutamate receptors, *grik1b*), also of auxiliary proteins of glutamate receptors of the AMPA-subtype (cornichon and pentraxin, Greger *et al.* 2017) and protein kinases (*e.g.*, *mapk11*), probably to be used in these rapid androgen effects. Also, and as already mentioned, androgens can have oncogenic effects, and several of the reported DE genes for the KT-treated group are indeed associated with tumors (*e.g.*, phosphoglycerate mutase 1, cathepsin Z, ephrin, Pernicová *et al.* 2011; Beauchamp and Debinski 2012; Hitosugi *et al.* 2012) while others are involved in neuroprotection (*e.g.*, *mapk11*, Nguyen *et al.* 2005) or neuronal growth (limbic system associated membrane protein, Pimenta *et al.* 1995), supporting previous evidence for the opposition between neuroprotective and neuroendangering roles of androgens (Foradori *et al.* 2008).

For the T-treated group (30 min sampling time point), secretagogin, a tumor marker (Birkenkamp-Demtröder *et al.* 2005) is up-regulated, while programmed cell death 1 and death effector domain-containing 1, genes involved in apoptosis, are down-regulated (Inohara *et al.* 1997; Sharpe *et al.* 2007). However chromatin- interacting genes were up-regulated (barrier-to-autointegration factor-like protein (Oh *et al.* 2015), suggesting the existence of epigenetic mechanisms underlying an increase of plasma testosterone. Together these results suggest that KT and T play distinct roles in the regulation of brain molecular processes.

According to our working hypothesis the forebrain transcriptome changes described above should represent the transcriptomic responses to social challenges mediated by transient changes in androgens, and should be mediating winner effects, hence reinforcing territoriality in resident territorial males. Although, a characterization of the forebrain transcriptomic response to social challenges for tilapia is not available, a time course study of the transcriptomic response after short territorial intrusions in the three-spined stickleback has been published (Bukhari *et al.* 2017). In this study the authors, using the same sampling time points (30 and 60 min) used in our study, found several sets of genes whose expression profile changed in concert together, originating different gene clusters with different temporal expression patterns (Bukhari *et al.* 2017). Moreover, genes belonging to each cluster had a similar function (Bukhari *et al.* 2017). This work supports the hypothesis that multiple waves of transcription are produced in response to a social challenge, with a first genomic response more related to stimulus perception, followed by a second wave of genomic response responsible for the behavioral response, then recovering and finally adjusting future behavior (Aubin-Horth and Renn 2009; Bell and Aubin-Horth 2010; Clayton 2013). The existence of waves of gene expression in response to social interactions is also supported in the honey bee with a similar behavioral paradigm (Shpigler *et al.* 2017). Likewise, our results emphasize that gene expression is dynamic and that selecting only a single sampling time point may miss the peak of transcriptomic response since at 60 min after androgen administration very few genes were differentially expressed. In contrast, at 30 min post-treatment a significant wave of transcription has been detected with most of the DE genes being down-regulated, in line with the results of Bukhari *et al.* (2017), suggesting that individuals respond first by down-regulating brain activity and afterward up-regulating it. In order to assess to what extent the androgen-driven transient transcriptomic response is part of the transcriptomic response to social interactions we have further compared the DEG lists for each time sampling in the present study with those elicited by a territorial intrusion in sticklebacks for the same sampling points (DEG 30 min: telencephalon = 246, diencephalon = 120; DEG 60 min: telencephalon = 614, diencephalon = 523; Bukhari *et al.* 2017). Two main differences are immediately observed: (1) the waves of transcription induced by a social challenge in sticklebacks peak at 60 min, whereas in the present study they peak at 30 min in response to androgens, suggesting different temporal dynamics for each transcriptional response; (2) At each peak of transcription, social challenges elicit a higher number of DEG than androgens, suggesting that the transcription-mediated behavioral responses to social challenge are not exclusively mediated by androgens. Moreover, a comparison of ortholog DEG between the two experiments shows an almost absence of overlap (see Table S9 and File S2), with only 4 ortholog genes (desmoglein, myosin, keratin and one undescribed gene) co-expressed at 30 min and one (plexin) at 60 min. These data further suggest a high independence of the transcriptomic response to social interactions from an androgen mediation, which is difficult to reconcile with the established key role of androgens as mediators of the winner effect in tilapia, which occurs at a timeframe (2h post-interaction; Oliveira *et al.* 2009) compatible with the sampling times used in this study (30 and 60 min). At least two possible explanations are possible for this mismatch: (1) there are species specific responses and the use of the stickleback response to social interactions is misleading; (2) The mediator effect of androgens on the winner effect is not mediated itself by changes at the forebrain transcriptome level, and depend on other physiological mechanisms at other brain levels or even on muscle physiology. In this respect, another important aspect to highlight is that in our study, we focused on a large brain area, the forebrain, that encompasses most nuclei that make up the social decision making network. Therefore, we captured the overall response of this network to transient androgen changes but we did not provide detail on putative regional differences across this network in the neurogenomic state of each of its nodes. In another transcriptomic study conducted in male threespined stickleback, it was found that several genes were up-regulated in the diencephalon and down-regulated in other brain areas in response to a territorial challenge (Sanogo *et al.* 2012). These results confirm the idea that each brain region has its own distinct neurogenomic response, and even if the same genes are differentially expressed in different regions, they can have in fact opposite regulatory directions (Sanogo *et al.* 2012). Therefore, with ongoing methodological developments and the reduction of sequencing costs, future studies should gain from the characterization of the transcriptomic response of each of the brain nuclei that together make up the SDMN.

In summary, our findings suggest that a transient rise of circulating androgens, such as the one observed after social interactions elicits relevant transcriptional changes, that may be part of an integrative process of adjusting future behaviors and promoting adaptive and socially competent behaviors.
